# Sequencing and analysis of the complete mitochondrial genome of *Hippopus porcellanus*

**DOI:** 10.1080/23802359.2021.1914208

**Published:** 2021-07-26

**Authors:** Haitao Ma, Baolu Zhang, Yuehuan Zhang, Yang Zhang, Yanping Qin, Chenghui Han, Shixi Chen, Ziniu Yu

**Affiliations:** a Key Laboratory of Tropical Marine Bio-resources and Ecology, Guangdong Provincial Key Laboratory of Applied Marine Biology, South China Sea Institute of Oceanology, Chinese Academy of Sciences, Guangzhou, China; b South China Sea Bio-Resource Exploitation and Utilization Collaborative Innovation Center, Guangzhou, China; c Hainan Provincial Key Laboratory of Tropical Marine Biology Technology, Sanya Institute of Oceanology Chinese Academy of Sciences, Tropical Marine Biological Research Station in Hainan, Chinese Academy of Sciences, Sanya, China; d Key Laboratory of Sichuan Province, Conservation and Utilization of Fishes resources in the Upper Reaches of the Yangtze River, College of Life Sciences, Neijiang Normal University, Neijiang, China; e Oceanic Consultation Center, Ministry of Natural Resources of the People’s Republic of China, Beijing, China

**Keywords:** *Hippopus porcellanus*, Mitochondrial genome, phylogenetic relationship

## Abstract

In this study, the complete mitochondrial genome of *Hippopus porcellanus* was reported. The whole mitochondrial genome was 21,565bp in length with a typical mitochondrial genomic structure including 13 protein-coding genes, 23 transfer RNA genes, 2 ribosomal RNA genes and 1 control region (D-loop). Mitogenome base composition was biased toward A + T content, at 60.3%. A phylogenetic tree based on complete mitogenome sequences revealed that, *H. porcellanus* is closely related to *H. hippopus*, both of which belong to the genus *Hippopus*.

Giant clams (Bivalvia: Tridacnidae), the largest bivalve mollusks in the world, inhabit the shallow coral reefs of the Indo-Pacific region. Giant clams are not only important to coral reef ecosystems, but also constitute a significant food source in Asia and the South Pacific, and are in demand for the shell and aquarium trade (Keys and Healy 1999). These clams belong to the subfamily Tridacninae, which has two genera namely: *Hippopus* and *Tridacna* (Othman et al. 2010). Two species belong to Genus *Hippopus*: *Hippopus hippopus* Linnaeus 1758 and *Hippopus porcellanus* Rosewater 1982. In this study, we sequenced the complete mitochondrial genome of *H. porcellanus* to further investigate of the taxonomy and phylogenetic relationships of Tridacnidae by increasing the amount of available molecular data.

The specimen was collected from Sanya, Hainan province, China (N109.51, E18.21) by a local fisherman, and stored in Tropical Marine Biodiversity Collections of South China Sea (TMBC), Chinese Academy of Sciences, Guangzhou, China (specimen accession number: TMBC030713). The total genomic DNA was extracted following the modified CTAB DNA extraction protocol (Attitalla 2011), and followed by library preps and pair-end sequenced (2 × 150 bp) with HiSeq (Illumina, San Diego, CA). Approximately 5,987 Mb of raw data and 5,216 Mb of clean data were obtained, and de novo assembled by the SOAP de novo software (Zhao et al. 2011) with an average of approximately 310× coverage.

The mitogenome of *H. porcellanus* was 21,565bp in length (GenBank accession number MT755622), containing 13 protein-coding genes (PCGs), 23 transfer RNA genes (tRNAs), two ribosomal RNA (*12S rRNA* and *16S rRNA*) genes and a non-coding control region (D-loop). The mitogenome base composition of *H. porcellanus* was biased toward A + T content at 60.3% (26.4% A, 33.9% T, 15.0% C, 24.6% G). The 13 identified PCGs vary in length from 114 to 1,677 bp. COI, ATP8, ND5, ND2, ND4L, ND1 and ATP6 initiated with ATG as the start codon, while ND3 and COIII begin with TTG, ND4 begin with ATT, COII with ATA, ND6 with GTG and Cytb with ATC. Three types of stop codons were TAA (ND4, COII, COIII, ND4L, ND6, Cytb), TAG (COI, ATP8, ND5, ATP6, ND3, ND1) and TAT (ND2). The lengths of the 23 tRNA genes ranged from 58 to 74 bp, and all of the tRNA genes contained typical secondary structure. The *12S rRNA* gene was located between tRNA-Leu and tRNA-Second Leu, and was 922 bp long, while the *16S rRNA* gene was located between tRNA-Ile and ND1, with a length of 1,269 bp. A 2,605 bp control region (D-loop) was located between tRNA-Met and COII, with an A + T content of 54.7%.

A neighbor-joining phylogenetic tree of *H. porcellanus* with five other closely related species was constructed with the complete mitochondrial genomes using MEGA6 (Tamura et al. 2013) (Figure 1). The result suggested that, *H. porcellanus* is closely related to *H. hippopus*, both of which belong to the genus *Hippopus*.

**Figure 1.**
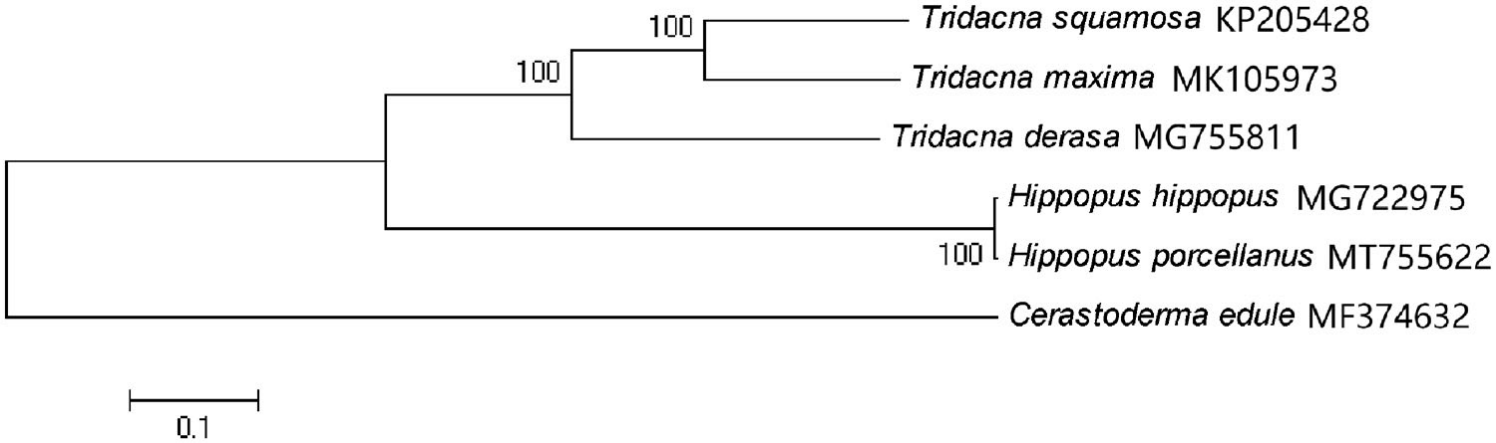
Neighbor-joining phylogenetic tree of *Hippopus porcellanus* and five other closely related species based on the complete mitochondrial genomes.

## Data Availability

The genome sequence data that support the findings of this study are openly available in GenBank of NCBI at (https://www.ncbi.nlm.nih.gov/) under the accession no. MT755622. The associated BioProject, SRA, and Bio-Sample numbers are PRJNA704757, SRR13827840, and SAMN18054235, respectively.
